# Simultaneous Fluorescein Angiography and Spectral Domain Optical Coherence Tomography Correlate Retinal Thickness Changes to Vascular Abnormalities in an In Vivo Mouse Model of Retinopathy of Prematurity

**DOI:** 10.1155/2017/9620876

**Published:** 2017-05-10

**Authors:** Olachi J. Mezu-Ndubuisi, Lauren K. Taylor, Jamee A. Schoephoerster

**Affiliations:** ^1^Departments of Pediatrics, University of Wisconsin School of Medicine and Public Health, Madison, WI, USA; ^2^Ophthalmology and Visual Sciences, University of Wisconsin School of Medicine and Public Health, Madison, WI, USA

## Abstract

**Background:**

Retinopathy of prematurity (ROP) is a condition of abnormal retinal vascular development (RVD) in premature infants. Fluorescein angiography (FA) has depicted phases (early, mid, late, and mature) of RVD in oxygen-induced retinopathy (OIR) mice. We sought to establish the relationship between retinal structural and vascular changes using simultaneous FA and spectral domain optical coherence tomography (SD-OCT).

**Method:**

63 mice were exposed to 77% oxygen at postnatal day 7 (P7) for 5 days, while 63 mice remained in room air (RA). Total retinal thickness (TRT), inner retinal thickness (IRT), and outer retinal thickness (ORT) were calculated at early (P19), mid (P24), late (P32), and mature (P47) phases of RVD.

**Results:**

TRT was reduced in OIR (162.66 ± 17.75 *μ*m, *n* = 13) compared to RA mice at P19 (197.57 ± 3.49 *μ*m, *n* = 14), P24, P32, and P49 (*P* < 0.0001). ORT was similar in RA and OIR mice at all ages (*P* > 0.05). IRT was reduced in OIR (71.60 ± 17.14 *μ*m) compared to RA (103.07 ± 3.47 *μ*m) mice at P19 and all ages (*P* < 0.0001).

**Conclusion:**

We have shown the spatial and temporal relationship between retinal structure and vascular development in OIR. Significant inner retinal thinning in OIR mice persisted despite revascularization of the capillary network; further studies will elucidate its functional implications in ROP.

## 1. Introduction

Retinopathy of prematurity (ROP) is a condition that impacts retinal vascular development (RVD) in preterm infants exposed to high levels of supplemental oxygen. Approximately 6–18% of childhood blindness cases in developed countries can be attributed to ROP [1]. In the United States alone, 14,000–16,000 preterm infants are diagnosed with ROP every year, and the worldwide prevalence is increasing [2]. ROP prevalence has been shown to be strongly correlated to prematurity, low birth weight, and oxygen therapy [3, 4]. Oxidative stress has been clearly implicated in the etiology of ROP [5]. Clinically, ROP is diagnosed and monitored subjectively with indirect ophthalmoscopy of premature infants and color fundus images using the International Classification of ROP (ICROP) [6]. Quantitative methods have not been established clinically.

Over the past two decades, ROP has been studied extensively with the mouse model of oxygen-induced retinopathy (OIR) using retinal whole mounts to quantify avascularity and neovascularization [7–9]. This model has been recently expanded to employ the use of fluorescein angiography (FA) [10] to quantify unique vascular features such as retinal arterial tortuosity, retinal vein dilation, and retinal vascular area or density [11], thereby outlining the phases of RVD. While abnormalities in revascularization and delayed vascularization following hyperoxia exposure have been a hallmark feature of ROP research, the structural changes in the retina because of these vascular abnormalities during this crucial period of retinal development have not been well understood. Although histology has been the gold standard for cross-sectional study of the retina in animal models of OIR [12, 13], it poses several challenges such as difficulty correlating vascular and structural findings, the need to sacrifice a lot of animals, and inadvertent artefacts from processing of devitalized tissues.

Optical coherence tomography (OCT) is an emerging non-invasive optical imaging method utilizing high resolution, and three dimensional (3D) structural contrast from the light backscattered from various ocular structures [14]. OCT has been gaining increasing use as a research and clinical diagnostic tool to provide a cross-sectional view of the retina and corresponding vasculature [2], thereby enhancing the study of ocular vascular diseases such as choroidal neovascularization [15] and retinal degeneration [16, 17]. Spectral domain optical coherence tomography (SD-OCT) has improved axial resolution over OCT and can provide further insight into intraretinal abnormalities [18]. SD-OCT is increasingly in use in clinical studies to examine retinal structure-function relationships in children with ROP [19, 20] and has revealed several retinal findings such as subclinical cystoid macular edema, subretinal fluid, absence or narrowing of the foveal pit, increased fovea thickness, and hypoplastic fovea [20–22]. OCT provides the capacity to monitor developmental, inherited, induced degenerative structural changes and therapeutic interventions in the same animal which could be widely applicable to preclinical studies [23]. SD-OCT use in OIR models has been limited due to a larger focus on vascular rather than structural changes in ROP, as well as the still emerging utility of in vivo imaging in OIR animal models. In our earlier studies, in vivo SD-OCT revealed retinal thinning in OIR mice compared to RA mice, more pronounced in avascular regions compared to hypovascular areas [24]. The spatial relationship of this observed retinal thinning is unclear, particularly whether it is localized to the inner or outer retinal layers. Additionally, it is unknown whether retinal thickness in OIR mice varies temporally during retinal vascular development.

This study sought to compare structural changes in the developing retina to vascular abnormalities at different phases of RVD with or without exposure to oxidative stress. While on its own, SD-OCT could provide details of the retinal structure and vascular network without the use of fluorescein dye; we have employed FA alongside SD-OCT in this study to match specific changes in thickness to changes in retinal vasculature at postnatal day ages corresponding to the phases of retinal vascular development.

## 2. Methods

### 2.1. Mice

C57BL/6J mice (Jackson Laboratory, Bar Harbor, ME) were reared in accordance with the Association for Research in Vision and Ophthalmology Statement for the Use of Animals in Ophthalmic and Vision Research. All experiments were performed according to approved protocols by the Institutional Animal Care and Use Committee at University of Wisconsin, Madison.

### 2.2. Simultaneous Retinal Spectral Domain Optical Coherence Tomography (SD-OCT) and Fluorescein Angiography (FA)

**Figure 1:**
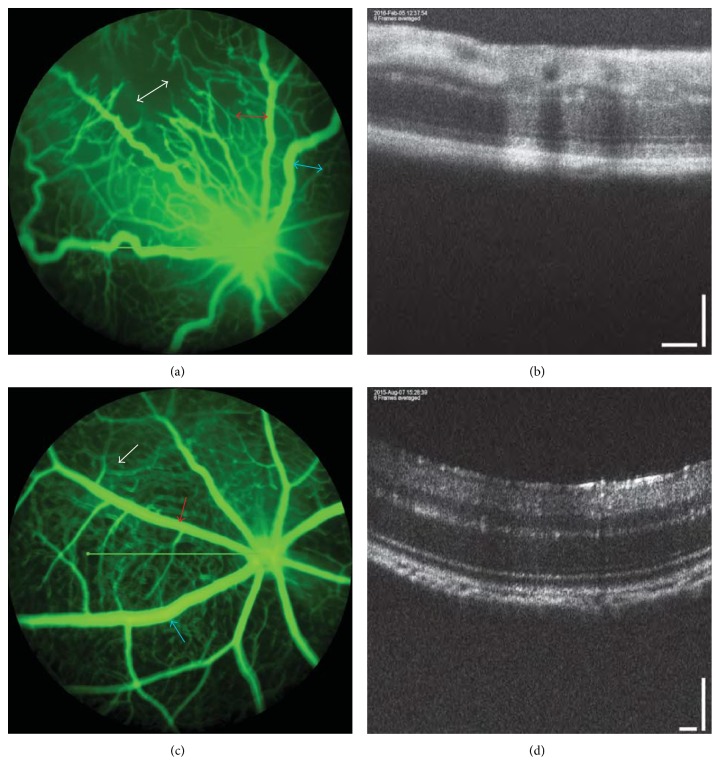
Fluorescein angiogram and corresponding spectral domain optical coherence tomography image at P24. (a1) FA of P24 OIR mouse depicting avascular retinal area in the superior retina (white double arrowhead), arterial tortuosity (red double arrowhead), and venous dilation (blue double arrowhead). (a2) Corresponding SD-OCT of the same P24 OIR mouse. (b1) FA of P24 RA mouse showing full capillary coverage with no avascularity in between major vessels (white arrow), nontortuous artery (red arrow), and normal width of vein (blue arrow). (b2) Corresponding SD-OCT of the same P24 RA mouse. Note: FA refers to fluorescein angiogram; SD-OCT refers to spectral domain optical coherence tomography; RA refers to room air.

Neonatal mice C57BL6/J mice were placed in a hyperoxia chamber with their nursing mothers at 77 ± 2% oxygen for 5 days (postnatal day 7 (P7) to P12) and then returned to room air (OIR mice) as described in a modification of an established OIR mouse model [7, 10]. The control group was reared in room air (RA mice). Mice were anesthetized with intraperitoneal ketamine (100 mg/kg) and xylazine (10 mg/kg), and their eyes were dilated with 1% tropicamide (Bausch + Lomb Inc., Tampa, FL), as previously described [11]. 100 mg/kg of 10% Sodium Fluorescein (AK-FLUOR, Akorn, Decatur, IL) was instilled through intraperitoneal injection prior to acquiring retinal images, followed by simultaneous FA and SD-OCT with the guidance of a bright-field live image using the Micron IV retinal imaging system (Phoenix Research Laboratories) in accordance with the manufacturer's image acquisition software (Figure 1()). The SD-OCT image shows retinal layers corresponding to a simultaneous image-guided bright-field live fundus image. The SD-OCT has a longitudinal resolution of 1.8 *μ*m, a transverse resolution of 3 *μ*m, imaging depth of 1.4 mm in tissue, and an imaging speed of 13,000 A-scans per second at 1024 pixels per A-scan. Available scan patterns in the SD-OCT machine are line, circle, and 3D volume. A line scan pattern was used in this study to obtain baseline cross-sectional thickness measurements. The bright green line was used to specify the spatial location of each OCT cross-sectional image and obtain 3D OCT scans of each mouse retina consisting of 1024 × 64 (horizontal × vertical) A-lines covering an area of 1 × 1 mm^2^. The line was positioned from the edge of the optic nerve to within 3 disc diameters in all RA and OIR mice.

#### 2.2.1. Segmentation of Retinal Layers

**Figure 2:**
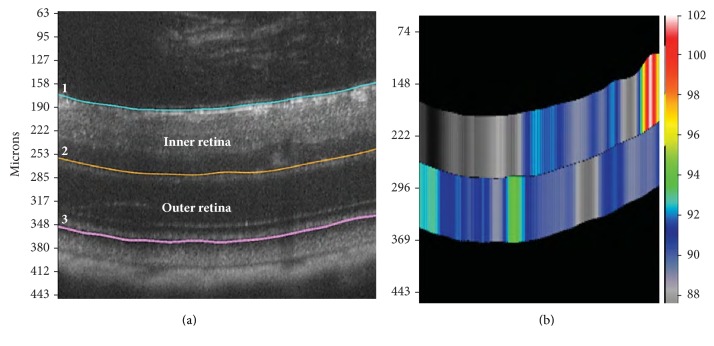
Spectral domain optical coherence tomography and corresponding thickness color map in P47 RA mouse. (a) Boundaries of the inner retina and outer retina. (b) Thickness color map for the SD-OCT generated from delineating the retinal boundaries. Line 1 was made at the superior boundary of inner limiting membrane (ILM). Line 2 was made at the lower edge of the IPL. Line 3 was made at the inferior boundary of the PRL. Lines 1 to 3: total retinal thickness is measured. Lines 1 to 2: inner retinal thickness is measured. Lines 2 to 3: outer retinal thickness is measured. Note: SD-OCT refers to spectral domain optical coherence tomography. Lines refer to linear segmentation lines.

Using the Insight software provided with the Micron IV SD-OCT imaging system, semiautomated segmentation was made of the total, outer, and inner retinal areas. The inner retina consists of nerve fiber layer (NFL), ganglion cell layer (GCL), inner plexiform layer (IPL), and inner nuclear layer (INL). The outer retina is comprised of the outer plexiform layer (OPL), outer nuclear layer (ONL), and photoreceptor layer (PRL). Three linear segmentation lines were made. Line 1 was outlined along the superior border of the inner limiting membrane (ILM). Line 2 was placed at the lower edge of the IPL. Line 3 was located at the inferior boundary of the PRL (Figure 2(a)). The software calculated the distance (thickness) in microns from line 1 to line 3 as the total retinal thickness (TRT), the distance from line 1 to line 2 as the inner retinal thickness (IRT), and the distance from line 2 to line 3 as the outer retinal thickness (ORT) (Figure 2(a)). A retinal thickness color map was generated for each OCT image (Figure 2(b)). We calculated the mean retinal thickness and standard deviations (SD) of the total, outer and inner retinal areas by averaging all the data points of the thickness maps/measurements automatically obtained from the semiautomatic tracing of the three layer boundaries.

### 2.3. Statistical Analysis

Two-way ANOVA comparisons were used to calculate differences in the OCT measurements at P19, P24, P32, and P47. Post hoc analysis was performed using Tukey's multiple comparisons for all pair-wise comparisons. For all experiments, a *P* value ≤ 0.05 was considered significant. Data are represented using mean ± SD.

## 3. Results

### 3.1. Retinal Thickness Changes Correspond to Phases of Retinal Vascular Development

**Figure 3:**
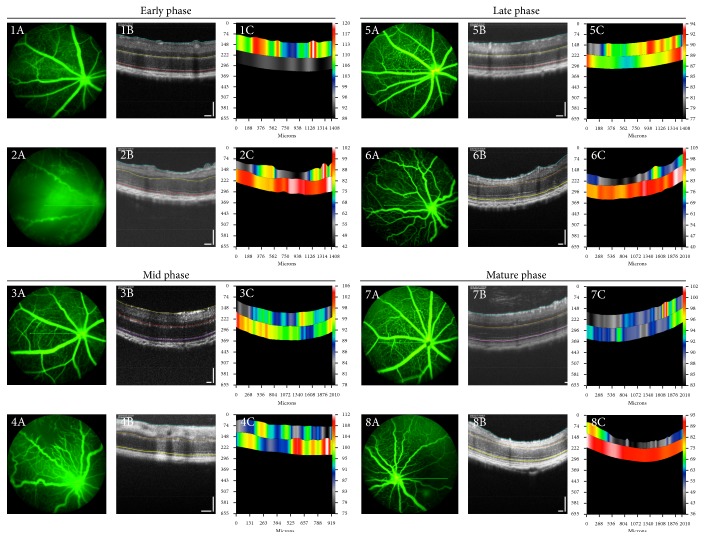
FA and SD-OCT at early, mid, late, and mature phases of retinal vascular development. (1A–C) represents P19 RA mice (early phase RVD); (2A–C) represents P19 OIR mice (early phase RVD); (3A–C) represents P24 RA mice (mid phase RVD); (4A–C) represents P24 OIR mice (mid phase RVD); (5A–C) represents P32 RA mice (late phase RVD); (6A–C) represents P32 OIR mice (late phase RVD); (7A–C) represents P47 RA mice (mature phase RVD); and (8A–C) represents P47 OIR mice (mature phase RVD). Note: SD-OCT refers to spectral domain optical coherence tomography; RVD refers to retinal vascular development. Numbering: 1 refers to P19, 2 refers to P24, 3 refers to P32, and 4 refers to P47. Lettering: A refers to FA image, B refers to SD-OCT image, and C refers to thickness color map.

**Figure 4:**
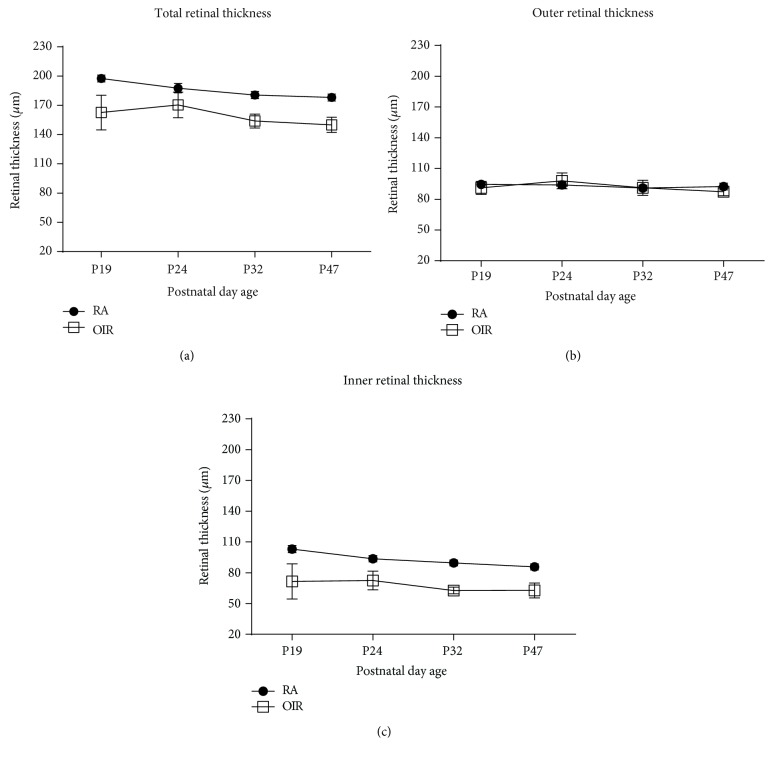
Retinal thickness (total, outer, and inner) at corresponding postnatal day ages of increasing retinal vascular development. (a) Total retinal thickness. (b) Outer retinal thickness. (c) Inner retinal thickness. Note: P refers to postnatal day; RA refers to RA mice; OIR refers to OIR mice.

As previously published, the early phase (P16–20) of RVD was characterized by marked dilation of veins and significant arterial tortuosity in OIR compared to RA mice [11]. There was circumferential obliteration of capillaries between the major vessels in OIR mice, but full vascularization in RA mice. Due to the absence of intraphase difference between the postnatal days in each phase of RVD in the original in vivo OIR method study [11], one day was selected as the representative of each phase. P19 in our study was the representative of early phase RVD in RA (Figures 3(1A)–3(1C)) and OIR mice (Figures 3(2A)–3(2C)). Mid phase of RVD (P22–27) was characterized by increased capillary regeneration and decreasing venous dilation compared to early phase, but still decreasing less than seen in RA mice of corresponding ages [11]. Though less tortuous than those in the early phase, arteries in the mid phase are still markedly more tortuous than RA counterparts. Mid phase RVD was represented in the current study by P24 FA images in RA (Figures 3(3A)–3(3C)) and OIR mice (Figures 3(4A)–3(4C)). Late phase RVD (P30–34) is characterized by sparsely dense capillary network despite full peripheral capillary regeneration, unlike the richly dense capillary network in RA mice of similar ages [11]. Although veins are equivalent in both RA and OIR mice in this group, tortuosity persisted unlike in RA mice. Our study, therefore, selected P32 to represent late phase RVD in RA (Figures 3(5A)–3(5C)) and OIR mice (Figures 3(6A)–3(6C)). In the mature phase of RVD (beyond P35), arterial tortuosity persisted with sparsity of capillary network and absent neovascular buds on capillary tips. In our study, FA in P47 mice represented changes in phenotype in the mature phase in RA (Figures 3(7A)–3(7C)) and OIR mice (Figures 3(8A)–3(8C)). Quantitative changes in arterial tortuosity, venous dilation, and capillary density have been clearly outlined in the previous studies [11] and were not repeated in this study. Our study displayed a correspondence between retinal thickness with increasing age in both OIR and RA mice and the changes in vascular phenotype seen in arteries, veins, and capillaries in the four phases of RVD described in original quantitative studies on the in vivo OIR method.  Table 1 shows the retinal thickness measurements: TRT (Figure 4(a)), ORT (Figure 4(b)), and IRT (Figure 4(c)) at the early phase (P19), mid phase (P24), late phase (P32), and mature phase (P47) of RVD.

### 3.2. Total Retinal Thickness Was Reduced in OIR Mice Compared to RA Mice

At P19, the TRT was reduced in OIR (162.66 ± 17.75 *μ*m, *n* = 13) compared to RA mice (197.57 ± 3.49 *μ*m, *n* = 14, *P* < 0.0001). OIR mice remained reduced at P24 (*P* < 0.0001), P32 (*P* < 0.0001), and P47 (*P* < 0.0001) compared to RA mice. With increasing age in the OIR mice, there was no change in TRT from P19 to P24 (*P* = 0.54), but TRT decreased significantly from P24 to P32 (*P* < 0.0001), while the thickness remained stable from P32 to P47 (*P* > 0.99). As the RA mice increased in developmental age, there was a physiologic decrease in TRT from P19 to P24 (*P* < 0.0001) and from P24 to P32 (*P* < 0.0001), but from P32 to P47, the TRT remained stable (*P* = 0.68) (Figure 4(a)) (Table 1).

### 3.3. Outer Retinal Thickness Remained Stable in Both OIR and RA Mice

There was no difference between the ORT in RA (94.51 ± 1.81 *μ*m) and OIR mice (91.06 ± 6.44 *μ*m, *P* = 0.07) at P19, P24 (*P* > 0.2), P32 (*P* > 0.99), and P47 (*P* = 0.14). In OIR mice, the ORT increased slightly (*P* = 0.0052) but was equivalent going from P24 to P32 (*P* = 0.66), as well as from P32 to P47 (*P* > 0.99). The ORT was unchanged in RA mice with each increasing age from P19 to P24 (*P* > 0.99), P24 to P32 (*P* = 0.24), and from P32 to P47 (*P* = 0.99) (Figure 4(b)) (Table 1).

### 3.4. Inner Retinal Thickness Was Reduced in OIR Mice Compared to RA Mice

IRT was significantly reduced in OIR (71.60 ± 17.14 *μ*m) compared to RA (103.07 ± 3.47 *μ*m, *P* < 0.0001) mice at all ages: P19, P24 (*P* < 0.0001), P32 (*P* < 0.0001), and P47 (*P* < 0.0001). There was no change in IRT in OIR mice going from P19 to P24 (*P* > 0.99), from P24 to P32 (*P* = 0.12), and from P32 to P47 (*P* > 0.99). IRT was decreased in RA mice from P19 to P24 (*P* < 0.0001) and from P24 to P32 (*P* = 0.02) but remained stable from P32 to P47 (*P* > 0.06). At P47, the IRT was still thinner in OIR compared to RA mice (*P* < 0.0001) (Figure 4(c)) (Table 1).

## 4. Discussion

ROP has been established as a condition of abnormal vascularization of the retina following preterm birth with potentially devastating functional consequences on vision. Premature infants have relatively immature retinas that are fragile and susceptible to insults that disrupt neurovascular development, leading to proliferative ROP [25]. Retinal thickness changes have been described with ex vivo histopathology in OIR mice, but the exact relationship between structural changes and vascular abnormalities in the developing retina have not been well elucidated. In vivo retinal imaging is an emerging field in preclinical studies of animal models of human retinal diseases; however, in vivo clinical use of SD-OCT has been generally limited to the assessment of macular volume and foveal thickness with limited attention to the structure of the rest of the peripheral retina, where significant ROP can occur. Our use of simultaneous FA and OCT in this study provides more detailed information about structural changes occurring in the retina at the same time as vascular abnormalities in ROP in the retina. Studying the relationship between angiogenesis and cytostructural development in the OIR mice is critical to understanding the pathophysiology of ROP.

Overall thinning of the retina in OIR mice was noted compared to RA mice, which is supported by a prior finding of reduced total retinal thickness in OIR mice [24]. In human studies of diabetic retinopathy, which shares a similar pathogenesis with ROP, there are reports of thinning of the ganglion cell and inner plexiform layer in the periphery [27]. Our findings differ from the findings of other studies that found increased total retinal thickness, specifically in the GCL and ONL, in premature infants with no ROP and spontaneously regressed ROP compared to term counterparts [19]. In our study, the outer retina in both OIR and RA mice maintained similarly uniform thickness throughout the retinal vascular development, despite hyperoxia exposure in OIR mice in our study [28], which differs from a report of thinning of ONL in OIR rat [12]. This is consistent with a study showing significant loss of TRT in OIR mice due to reduction in thickness of the IPL and INL, while the ONL remained stable [26].

Though the mouse is born at term, the retinal vessels develop postnatally and are therefore susceptible to oxidative stress and inflammatory insults from external stimuli. In the present study, from P7 to P12, hyperoxia exposure causes obliteration of developing retinal vasculature, evident in P19 and P24 FA images (Figure 3()), and when the mice are returned to room air, the relative hypoxia also can cause a reperfusion injury from revascularization attempts. The immature retina of the mouse is therefore susceptible to hypoxic-ischemic damage [29]. The ganglion cells in the rat inner retina have been shown to be prone to cell death in the setting of hypoxia, which can cause retinopathy [30]. Further studies are needed to correlate our findings of reduced inner retinal thickness with SD-OCT in the present study with necrosis or apoptosis of the ganglion cell layer.

Retinal thickness changes observed in our study appear to correlate with the phases of RVD described previously [11]. The Muller cells, found in the inner nuclear layer, are associated with retinal blood vessels [31] and are the major source of vascular endothelial growth factor (VEGF) in the retina [32, 33]. VEGF is a well-studied potent mediator of angiogenesis in the retina [34, 35], which if suppressed would inhibit vessel growth and if increased would promote retinal neovascularization [35]. Our finding of reduced capillary density in the mature phase of OIR mice could be a result of this thinning of the inner retinal layer. Evaluation of VEGF levels in the retina or localization of VEGF in the inner retinal layer was beyond the scope of this study. Persistence of retinal thinning in the inner retina in adult mice in our study is consistent with the sparsity of the capillary network and persistence of tortuosity described in the mature phase of RVD, in the original study of in vivo OIR quantitation methods [11].

Clinical correlation of structural changes seen in SD-OCT in OIR mice to premature infants with ROP is challenging because the mouse retina does not have a fovea. Foveal changes have been the main focus of SD-OCT clinical studies, rather than overall retinal thickness abnormalities [20, 21, 36]. Children with regressed ROP have increased foveal thickness, altered foveal pit shape, reduced avascular zone, and cystoid macular edema [20, 21, 36]. In a clinical study of 114 full-term control infants and 60 preterm infants (with and without ROP), the preterm patients, both with and without treated ROP, did not show any macular or foveal thickness or structural differences compared with the term group; however, early preterm children with previously treated ROP had flatter RNFL profiles with reduced thickness in superior and nasal quadrants, a thicker temporal quadrant, and a thinner average and minimum GCL-IPL complex [37]. Their finding of thinner GCL-IPL is in agreement with our findings of a reduced thickness in the inner retinal layer.

The limitations of this study relate to the semiautomated calibrations of the boundaries of the outer and inner retina with the manufacturer's software, which could generate minor subjective variations. Retinal thickness scans in this study were done with lines not circles, starting at the edge of the optic disc, and included both large vessels and capillaries along the path of the line, which could have affected the determination of thickness measurements in both RA and OIR mice. Therefore, in this study design, avascular portions were not measured separately from vascular portions, but rather, both may be included within the line scan, in order to facilitate more accurate determination of baseline thickness measurements and a more physiologic phenotype of the OIR mice retina at various phases of RVD. Another limitation was that different mice were used at each postnatal day tested, and this was not a repeated longitudinal study on the same mouse throughout development. The experiment was designed in this way to establish reliable baseline values for retinal thickness without the bias that could be inadvertently created by mouse fatigue with repeated testing, slow clearance of fluorescein from the eye, and poor imaging visualization due to cataracts formed by prolonged light exposure and retinal imaging. This study did not evaluate the etiological factors responsible for inner retinal thinning, such as histological localization of apoptosis or VEGF expression in the retinal layers. Finally, clinical correlation of our findings would not be applicable to foveal changes due to the physiologic absence of fovea in mice.

## 5. Conclusion

Our study shows that in ROP vaso-obliteration from hyperoxia exposure and subsequent vasoproliferation from revascularization in relative hypoxia result in cytostructural alterations that correspond to persistent loss of capillary density despite full retinal vascularization and arterial tortuosity. We have elucidated the physiologic spatial and temporal relationship as well as natural progression of retinal structural and vascular changes in OIR using simultaneous in vivo SD-OCT and FA. Total retinal thickness was significantly decreased in both neonatal and adult OIR mice from postnatal oxygen exposure during RVD compared to RA mice. Retinal thinning in OIR mice occurred specifically in the inner retina during early phase RVD (P19) and persisted in mature phase RVD (P47). RA mice had a physiologic decrease in inner retinal thickness with age up to P32, and then thickness was preserved from late to mature phase RVD. Our study suggests significant thinning of the inner retina (Muller cell, ganglion cell, or inner plexiform layer) in ROP. The functional significance of inner retinal thinning despite full revascularization in ROP needs to be further investigated. Further in vivo SD-OCT studies in OIR are needed to aid the understanding of the pathophysiology of ROP beyond microvessels to encompass structural changes, their functional implications, and correlation to neurovascular abnormalities. This will enable identification of the cellular signaling mechanisms involved, which may lead to potential new therapies for ROP.

## Figures and Tables

**Table 1:** Comparison of retinal thickness at different postnatal day ages.

Comparison of retinal thickness (total, outer, and inner)							
Postnatal day (P)	RA		*n*	OIR		*n*	*P* value
Total retinal thickness (*µ*m)							
P19	197.5724	3.49458	13	162.6646	17.74571	13	<0.0001
P24	187.6141	4.842831	20	170.3827	13.06806	21	<0.0001
P32	180.5107	3.674493	16	153.8795	7.093813	16	<0.0001
P47	178.0933	3.573367	15	150.0147	7.850246	13	<0.0001
Outer retinal thickness (*µ*m)							
P19	94.50678	1.808792	13	91.05743	6.439356	13	0.6739
P24	93.94167	2.805339	20	97.93496	7.6444	21	0.2054
P32	90.9426	2.800284	16	91.19257	7.367622	16	>0.9999
P47	92.39932	3.301538	15	87.24262	3.752146	13	0.1447
Inner retinal thickness (*µ*m)							
P19	103.0656	3.466552	13	71.6072	17.13845	13	<0.0001
P24	93.67243	2.988985	20	72.44776	9.084769	21	<0.0001
P32	89.56806	2.802428	16	62.68689	3.19307	16	<0.0001
P47	85.694	2.984063	15	62.77211	7.274023	13	<0.0001
